# Model-Driven Approach for Body Area Network Application Development

**DOI:** 10.3390/s16050670

**Published:** 2016-05-12

**Authors:** Algimantas Venčkauskas, Vytautas Štuikys, Nerijus Jusas, Renata Burbaitė

**Affiliations:** Department of Computer Science, Kaunas University of Technology, Studentu 50-212, Kaunas LT-51368, Lithuania; vytautas.stuikys@ktu.lt (V.Š.); nerijus.jusas@ktu.lt (N.J.); renata.burbaite@ktu.lt (R.B.)

**Keywords:** Internet of Things, security and privacy, body area network, WNS, quality-of-service, BAN software design, model-driven approach

## Abstract

This paper introduces the sensor-networked IoT model as a prototype to support the design of Body Area Network (BAN) applications for healthcare. Using the model, we analyze the synergistic effect of the functional requirements (data collection from the human body and transferring it to the top level) and non-functional requirements (trade-offs between energy-security-environmental factors, treated as Quality-of-Service (QoS)). We use feature models to represent the requirements at the earliest stage for the analysis and describe a model-driven methodology to design the possible BAN applications. Firstly, we specify the requirements as the problem domain (PD) variability model for the BAN applications. Next, we introduce the generative technology (meta-programming as the solution domain (SD)) and the mapping procedure to map the PD feature-based variability model onto the SD feature model. Finally, we create an executable meta-specification that represents the BAN functionality to describe the variability of the problem domain though transformations. The meta-specification (along with the meta-language processor) is a software generator for multiple BAN-oriented applications. We validate the methodology with experiments and a case study to generate a family of programs for the BAN sensor controllers. This enables to obtain the adequate *measure* of QoS efficiently through the *interactive adjustment of the meta-parameter values and re-generation process* for the concrete BAN application.

## 1. Introduction

In recent years, it has been a very broad discussion about the Internet of Things (IoT) and IoT-related applications in healthcare [1,2]. A significant part of those concern Body Area Networks (BANs) [3]. These, in fact, are Wireless Sensor Networks (WSNs). In the case of the IoT therefore, the BAN (also Wireless BAN) represents a node of the IoT. The following explain BANs’ popularity: (1) in general, the monitoring of human health is the predominant factor; (2) the BAN can cover different sub-domains of a human’s activity. We can assume, for instance, that the BAN cell, as a node of the IoT, is near to the human (e.g., it is in his/her pocket or bag) and the cell’s units such as sensors are mounted on the body to collect data for evaluating the human’s health state. The human, for example, may be a patient, a tourist climbing in the mountains, a worker operating in a harmful environment, *etc.* for whom the monitoring of the health state is highly important. Both the cell and human represent a *thing* of the IoT. The BAN, like many other IoT-based applications, has to operate under stringent constraints when transferring data over the network.

Typically, security/privacy, energy-awareness and environmental factors represent the major constraints in such applications. The first is due to the possibility that the data can be launched and changed, e.g., during the transfer sessions. The second is due to the use of battery-charged devices within the network. The third is due to the noises that influence data transfer. Those factors are highly related and extremely complex in their own way. For example, there are a variety of communication protocols to ensure different levels of security. The more complex a protocol is (meaning the complexity of the encryption algorithms used), the more energy is required to ensure the required level of security. The same is true of environmental noises. All those factors, when considered together, predefine the quality of service (QoS) of the application. Therefore, QoS should appear as a basic non-functional requirement in designing the IoT applications. As our literature review shows, security and energy issues are seen as predominant factors in healthcare and other WSN-related applications. As those applications cover multiple aspects, we need to introduce the reader to the whole spectrum of their heterogeneity.

In this paper, we consider the BAN prototype as a node of the IoT with respect to the QoS requirements. The BAN itself consists of *internal nodes* (sensors and actuators). Typically, the number of nodes ranges from a few to a dozen nodes. That depends on the type of each BAN application (e.g., runners require less sensors than a patient). The nodes may be combined into groups to cover different aspects of the same application, or even different but related applications. Therefore, the functionality and structure of a node can differ significantly, depending on the requirements of a particular application. On the other hand, there might be identical nodes (e.g., for ensuring better performance, higher reliability, *etc.*). Sensors are for collecting and sending data from the body. Actuators are for reacting to the changes and on this basis, making a decision by an actor. Both may also possess the smart features such as the computation and decision making capabilities.

The hardware parts of a BAN (such as smart sensors and actuators) are rarely made from scratch. Most likely, they are obtained as reusable components from commercial suppliers. That is possible due to the technology advances (standardization, high-level of automation, *etc.*). However, this typically is not the case with regard to software, because it is highly dependent on the particular requirements of an application. Typically, the software of a particular IoT application is unique, though it also may have common features with other applications, such as managing and control facilities for transferring data. For IoT applications, software is regarded as a stem of the whole. It might be either embedded into sensors, or be used as an external component [4]. Therefore, the software development process is hard. The non-functional requirements such as for security and energy, along with the functional requirements, make the software development even challenging and specific as compared to multiple other software applications.

As the complexity of systems is steadily increasing, software development approaches should rely on successful ones used in the industry, such as software Product Line Engineering (PLE). The PLE approach is defined as a methodology to develop a family of related products in an efficient way, taking full advantage of the products’ commonality and variability aspects [5]. It is also concerned with the use of the *variability management* [6], high-level *feature modeling* [7] and *feature model* *transformations that* typically are defined as model-to-model or model-to-program transformations [8]. Therefore, the model-driven software development approach is centered on the use of models and their transformations [8].

In IoT applications, due to their novelty and specificity, model-driven approaches are not yet exploited in full to ensure better quality, higher productivity, more flexible adaptability and reuse. Therefore, the aim of the paper is to analyze and disclose the potential of the model-driven approach for designing software for BAN-oriented applications. Currently, the main problems in designing those applications are as follows: An insufficient extent of automation, inadequate capabilities for process reuse, integration and adaptation. Furthermore, so far designers have largely ignored the multiplicity and synergy of non-functional requirements in designing their systems.

Though in general the model-driven approach is well known and widely used, our contribution is two-fold: (1) We have seamlessly integrated QoS, representing the important ingredients of BAN uniformity, into the full development cycle that includes the BAN prototyping, requirement statements, modeling and implementing the application software through model transformations; (2) Our approach results in the creation of *an* *executable meta-specification* to automatically generate the essential part of software for a family of related BAN applications. That enables one to significantly enhance the BAN development for multiple applications along with the semi-automatic adaptation to fit the QoS requirements for the concrete BAN application.

The methodology we discuss here has three significant parts: (1) specification of requirements at the higher level of abstraction by feature models to specify the problem domain (*i.e.*, BAN); (2) introduction of generative technology (*i.e.*, meta-programming as a solution domain that is also presented by the feature model); (3) seamless integration of the problem and solution domains through model transformations to achieve the goals of automation, adaptation and reuse. Though all the parts are for the use by IoT software developers, part (1) may also be useful for the much larger community, such as IoT domain policy makers, IoT and BAN researchers and practitioners.

The paper structure is as follows: In Section 2, we analyze the related work. In Section 3, we formulate research objectives and tasks. In Section 4, we describe the requirements and the basic idea of the approach. In Section 5, we present the methodology and its background. In Section 6, we analyze the case study and experiments we carried out for the BAN-oriented applications. In Section 7, we discuss and evaluate the results. Finally, in Section 8, we provide conclusions on the results.

## 2. Related Work 

We categorize the related work into parts A and B. In part A, we analyze the general trends in IoT research and healthcare applications, focusing on security, privacy and energy challenges within the IoT, WSNs and WBANs. In the part B, we provide analysis of modeling and application aspects to support the computational issues of the IoT, WSNs and WBANs.

### 2.1. Part A: Analysis of the Security, Privacy and Energy Challenges within the IoT, WSNs and Healthcare

As stated in the Introduction, the trends, vision, challenges and problems imposed by the IoT are discussed in [1,2]. Though there are multiple IoT applications, healthcare applications are at the focus in this regard. For example, Kumar *et al.* [9] discussed the challenges of security and privacy in healthcare applications, an integral part of which are wireless medical sensors. The authors also defined the relevant requirements of security for these applications and formulated some open research problems.

In mobile healthcare services, the accurate detection of an emergency situation and its notification are critical to chronic patients’ life. Chun *et al.* [10] described the automatically detected emergency condition of the individual bio-data collected from wireless BANs using a message flow diagram based on a personalized emergency policy. Wan *et al.* [11] analyzed cloud computing applications in healthcare systems and possible cloud-related WBAN solutions. They also considered approaches for transmitting vital data to the cloud by using energy-efficient routing, for allocating cloud resources, as well as semantic interactions and data security schemes.

Oh *et al.* [12] dealt with a lightweight security system for the IoT with regard to the limited computing resource availability and memory capacity. The system exploits an innovative malicious pattern-matching engine. The provided experiments showed that the proposed system offers scalable performance for a variety of patterns. Choi *et al.* [13] presented a user-driven environment which is designed for modeling and creating IoT services. As the IoT objects can acquire huge amounts of data from different contexts (home, office, industry, body), data fusion processes are applied to form more complex information structures.

Hughes *et al.* [14] reviewed the existing investigation in the field of WBAN engineering, including protocol adaptation and energy effective cross-layers by highlighting how the existing solutions resolve the various issues specific to remote permanent healthcare monitoring. Chen *et al.* [15] gave a holistic review of security challenges in sensor networks divided into the following classes: cryptography and key distribution, secure routing and location security, attack detection and prevention, secure data fusion, and other security challenges. The authors also summarized the methods and techniques used in these classes. Slavin *et al.* [16] introduced the security requirements as patterns to represent reusable security technologies that software developers can use to improve the security of their systems. The authors proposed a novel approach that combines an inquiry-cycle based method with the *feature diagram notation* to check only relevant models and to select the most suitable models for the situation.

Selimis *et al.* [17] emphasized the importance of ensuring and protecting patients’ sensitive data obtained in WBANs and proposed a new microcontroller architecture in order to improve the security and reduce the energy consumption of the system. In the context of using WBANs, Crosby *et al.* in a survey paper [18] examined the following issues: Monitoring and sensing, power efficient protocols, system architectures, routing and security and formulated some open research topics. Jing *et al.* [19] discussed the cross-layer heterogeneous integration and the security problems of the IoT, analyzed solutions to them and compared security challenges between the IoT and traditional networks, and also highlighted emerging security challenges of the IoT. Hosseini-Khayat [20] considered the security—energy relationship at the level of wireless implantable medical devices by providing the lightweight encryption protocol that can be used to implement on the very low power ASIC chips.

Energy consumption is the main challenge in WSN as well as in the IoT. Therefore Zhou *et al.* [21] presented the energy models of the device major components: processors, communication modules, sensors and actuators. An IoT device energy model can exactly disclose the energy consumption of the sensor unit. Rani *et al.* [22] considered the green networked IoT and addressed energy consumption challenges by proposing a novel implementation scheme. This scheme consists of: (1) a hierarchical network design; (2) a model for the energy efficient IoT; (3) a minimum energy consumption transmission algorithm to implement the optimal model.

### 2.2. Part B: Analysis of Modeling to Support Implementation Issues of the IoT, WSNs and WBANs

Schmidt *et al.* [23] proposed an approach to design sensor node models based on ordinary measures. They provided a case study where models are also incorporated in a modeling environment in the runtime framework of a model driven design. Measurements on an experiment implementation demonstrated a decrease in energy consumption as compared to an application without energy saving technique (*i.e.*, without the use of proposed models).

Ryu *et al.* [24] proposed an *integrated semantic service platform* to support ontological models in different IoT-based service domains of a smart city, addressing three main issues for supplying complex semantic services along with IoT systems: semantic discovery, dynamic semantic presentation, and semantic data storage for IoT systems. Vu *et al.* [25] described an extensible modelling environment to simulate WSNs. Particularly, the suggested modeling tools have facilitated the investigation of the sensor nodes communication security. The simulator’s main components are: network topology model, key establishment protocol, and adversary model for node capture, network analysis tools, and a graphical user interface to facilitate the rapid simulation, visualization, and analysis of WSNs.

Ortiz *et al.* [26] considered the runtime variability, a main technology for Dynamic Software Product Lines (DSPLs), as certain applications demand reconfiguration of system features and execution plans at runtime. In this emerging research work, the authors addressed the problem of dynamic changes in feature models in sensor networks product families, where the nodes of the network demand dynamic reconfiguration at post-deployment time.

The functionalities provided by objects of the IoT can be viewed as ‘real-world services’ as they provide a near real-time state of the environment [27]. Therefore, this paper described a semantic modeling method for different units in an IoT framework. It also discussed how the model can be included in the IoT framework by using automated association techniques with physical units and how the data can be obtained using semantic search and reasoning techniques.

Fajar *et al.* [28] performed feature modeling for analyzing commonality and variability among the applications in terms of their features and visualize analyzed commonality and variability in a tree-form diagram. The feature model provides a comprehensive view of the Wireless Sensor/Actuator Network (WSAN)-based agriculture system and helps agriculture domain experts and software engineers communicate intuitively. Ruiz-Zafra *et al.* [29,30] described a model-driven approach for developing high-level software interfaces that allow developers to easily interact with wearable devices or BAN sensors and to reduce risks and development efforts.

In feature-based modeling there is an evident shift from static to dynamic modeling with the focus on context modeling. Examples are the collaborative context features modeling of critical systems [31], dynamic feature modeling of recommender systems [32], decision-making using dynamic feature models [33], to name but a few. Capturing and representing this high-level dynamic context in creating the IoT systems is also a big challenge. Context-awareness is extremely concerned with the high degree of variability and its dynamic mode (changeability). The context variability modeling techniques, as Venckauskas *et al.* analyzed in [34], are focused on the sensor (mainly) and middleware layers (partially). In the variety of the IoT applications, the non-functional requirements (such as human behavior models, information security at the application level or pure technology, such as the level of the energy consumption, *etc.*) can be treated as the application-level context. Therefore, the feature modeling at this level can be seen as a relevant technique.

Though the analysis provided here is by no means comprehensive, we are able to formulate the following main findings (1) WSNs, as predecessors of the IoT, and BAN-oriented applications, as an important segment in healthcare, and are at the focus of attention now; (2) typically, the factors (security-privacy, energy savings and environmental factor) within IoT components (such as WSNs and BANs) are widely discussed, but considered separately due to their complexity or due to the need to substantially improve existing solutions. This, however, is not enough for building IoT applications. For BANs, for example, a major challenge is not so much to take into account a separate factor, but rather the synergistic effect of those factors on functionality and quality of such applications. As those applications are relatively new, there is a lack of approaches that focus on such a vision, though the understanding of that problem (typically it is identified as QoS) can already be found in the literature; (3) so far, to our best knowledge, developers either design BAN-oriented software for the concrete application, or use model-driven approaches restrictively, when one expresses models by features; therefore, the capabilities for design automation and reuse are limited. This limitation, in fact, motivates our approach; (4) another big issue is how to bridge the gap between the QoS requirements and IoT applications and bring QoS into the system design process. Therefore, little is known in the analyzed literature on how to close the gap, though there are a few works in the IoT domain that use the model-driven development in designing the IoT systems. Those findings enabled us to motivate our research tasks as described below.

## 3. Research Objectives and Tasks

As stated in the introduction, our research object is BANs. Since we consider the object as a node of the IoT for multiple applications, multiple aspects are to be taken into account. Typically, the main concern of BAN-related applications is to build: (1) the adequate software as effectively as possible and (2) this software should be as adaptable and reusable as possible to fit the multiple requirements and the multiple use cases. Therefore, the objectives of this research are to introduce a model-driven methodology that enables one to enhance the processes in developing software for BAN applications. The main focus of our research is the extended capabilities for automation, *i.e.*, creating a generic specification (through model transformations) to derive a family of application programs for possible adaptation for a concrete use case). The research questions (RQs) we address in this work are:

RQ1. Creating consistent feature models for the problem domain (PD, *i.e.*, BAN).

This RQ should be dealt with so that it would be possible to specify multiple requirements in two modes (as separate entities and their relationships and as the unified measure of QoS for the non-functional requirements).

RQ2. Representing the *solution domain* (SD, *i.e.*, meta-programming) also by the feature model and then specifying transformation rules for connecting both the PD and SD models.

This RQ should be dealt with so that the *mapping* of the PD model onto the SD model would be possible as well as the subsequent model-to-meta-program transformation resulting in the creation of the meta-specification for generating BAN applications.

RQ3. Validating the results of RQ1 and RQ2 and overall methodology proposed.

This RQ is intended to be solved through experiments and a case study.

The novel parts of our approach are as follows: (i) the measure of QoS within the feature models to specify a multiplicity of the predefined requirements (for security, energy, *etc.*) for a family of possible BAN applications, though in general the model-driven approach is well known and widely used [35]; (ii) the meta-specification we describe in this paper has a distinguishing property—an interface combined with the feedback—to adapt the QoS characteristics to generate a particular BAN application program on the user’s demand, though the use of heterogeneous meta-programming as a part of the model-driven approach is also well known (see, e.g., [36,37]); (iii) furthermore, the model-driven methodology as applied to BAN introduces systematization in designing software, brings capabilities for automation and adaptation.

With regard to the model-driven approach, we have selected the feature models due to the following reasons: (a) they are both human readable and machine interpretable, *i.e.*, they have graphical and textual representation; (b) they enable one to express the problem domain variability well (typically through variation points and variants); (c) there is a dozen of examples and use cases of successfully implementing the models in practice; (d) it is possible to discover the relationships between feature concepts and meta-programming concepts.

## 4. Requirements for Implementing IoT-Oriented BAN Applications

The BAN-oriented sensor network is concerned with the data collecting from a human’s body, manipulating the data and *transferrin*g/*accepting* the information *to*/*from* the application level. There might be a variety of the BAN applications related to monitoring the human’s health state (the two terms BAN and WBAN are synonymous, but hereinafter we will use the first). The sensor network can be seen as a sub-net of the IoT. Those applications are indeed complex, because we need to take into account a variety of issues such as device heterogeneity, scalability, energy-optimized solutions, security, *etc.* [1]. The human, along with the series of sensors, are *things* connected via the Internet to a remote station for monitoring or making a decision. The top level implementing the BAN application is at the remote station. In the case of the patient, for example, the station is a hospital or other treatment institution. In the other case, the station can be the remote environment monitoring the human’s state within the harmful production area, *etc.* The remote thing is able: (1) *to monitor* the health state; (2) *to control* changes of the measured data *and provide* the adequate reaction; (3) *to react* to the physical changes within the cell hardware (e.g., significant reduction of the battery’s energy); (4) *to send messages* to the remote station informing about any changes in the human’s state and physical changes in the environment in order for it to be possible to make adequate decisions.

It is possible to estimate the health state by measuring body data such as temperature, blood pressure, pulse, *etc*. The data comes from the sensors mounted on the human’s body and connected with the core cell, called Smart Sensor Controller. Note that there might be a specific kind of the smart sensor, called smart actuator, which is responsible not for manipulating measured data, but for performing some physical action such as injecting insulin into the human’s body, *etc*.

Further, we introduce and define some terms related to the BAN-oriented sensor network applications. By a *node of the IoT*, we mean a patient with smart sensors mounted on his/her body along with the smart sensor controller for the data acceptance, decision making and controlling data transfer in both directions: From and to smart sensors and from and to the application level.

**Figure 1:**
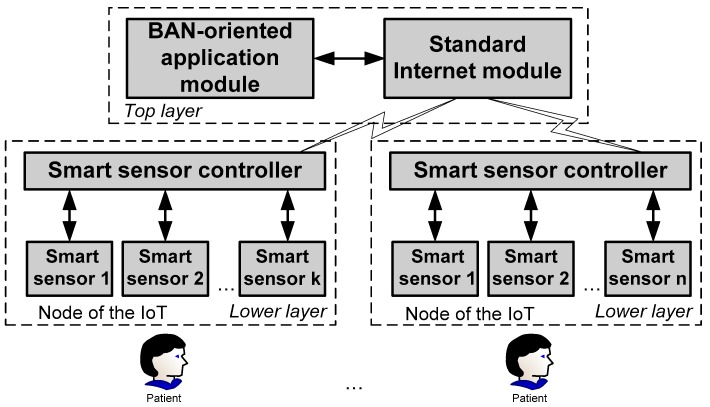
Two-layered sensor-networked IoT model to support BAN-oriented applications.

*Definition 1.*  *BAN* is a net of the IoT nodes. *BAN-oriented IoT model (prototype*) is the two-layered architecture containing the standard Internet and application modules at the top layer and the IoT nodes at the lower layer (see Figure 1).

As we consider the software development for such BAN-oriented applications, the specific requirements are to be stated in the first place. We focus on *non-functional* and *functional* requirements at two levels (generic specification and implementation). The first stands for specifying of a larger family of the related applications. With regard to non-functional requirements (security/privacy, energy *etc.*), we specify them as *quality of service* (QoS) [3] since a unified measure is more convenient for the interpretation and implementation. The second level stands for representing more details needed for the implementation we describe later.

*Definition 2.*  QoS is a trade-off of the available *energy resource*, *system performance, security level* and *environmental factor* to ensure *the acceptable degree* of the IoT node functionality for the user (see also relationship (1) and *Definitions 3 and 4*).

*Definition 3.*  Security level is the privacy/security requirement identified as a value taken from the set {*U*, *SU*, *R*, *C*, S, *TS*}, where the elements of the set are *fuzzy variables* having the following meaning: *U*—Unprotected, *SU*—Secure Unprotected, *R*—Restricted, *S*—Secret, TS—Top Secret [35].

The security level depends on the communication protocol and the encryption algorithm used within the data transfer protocol. For example, the use of the most effective encryption algorithm ensures the top security (TS).

By the system performance, we mean the clock frequency of the processing unit either within a smart controller, within a smart sensor, or both. The decrease of the frequency highly impacts on the energy needs [36].

With regard to the definition of QoS, there are a few important aspects to consider. First, each constituent of the measure has a set of the possible values. Those values are either predefined in advance such as security levels [35], or are defined during the operation on-the-fly dynamically such as the battery’s energy amount. Second, we assume that it is possible to change the clock frequency of the processing unit so that to make an impact on its performance (if needed, for example, due to the significant drop in energy). Third, each constituent is supplied with the fuzzy variable to estimate the weight of the constituent by equalizing its role in the measure of QoS [32]. Fourth, all constituents are highly dependable in that the change of the value of one constituent causes the need to adapt the value of another. Finally, it is worth to note that the energy is the most important attribute influencing to QoS and the whole functionality of the node.

Therefore, we can speak about the measure *M* of QoS, identified as *M*(QoS). It is varying over time too. We can estimate the boundaries of this variation and write the following relationship:

*M*(QoS)_min_ ≤ *M*(QoS)_c_ ≤ *M*(QoS)_max_ (1)

We obtained the measure by experiments described in [34,38,39]. In [39], it is treated as a Pareto-optimal solution. However, in the case of developing a software application, it is more relevant to express the measure at the top level through the needed security level (see *Definition 3*).

*Definition 4.*  An acceptable degree of the functionality of the IoT node is the measure *M*(QoS)_c_ satisfying the relationship Equation (1) (where *M*(QoS)_c_ is the current value of the measure *M*).

The main functional requirement is to ensure *uninterruptable sessions* of the data stream from the smart sensors to the smart controller and from the latter to the remote station. Therefore, the smart sensor’s functionality is at the focus. The control programs designed under the strict non-functional requirements predefine the functionality. Further, due to its complexity we investigate not the whole system, but its lower-layer part identified as a node of the IoT (see Figure 1).

Therefore, we have listed functional and non-functional requirements as generic ones along with a generic two-level IoT model to investigate and implement BAN applications. More specifically, functional requirements include: (i) identification of the type and the number of sensors and actuators required to satisfy the BAN needs; (ii) definition of their functions; (iii) selection of communication protocols and their operation modes; (iv) combining the selected items into a BAN-oriented WSN. The list of non-functional requirements is expressed through QoS to uniformly define the impact of security, energy, and environmental factors.

## 5. Methodology and Background

### 5.1. Basic Terms and Background

**Figure 2:**
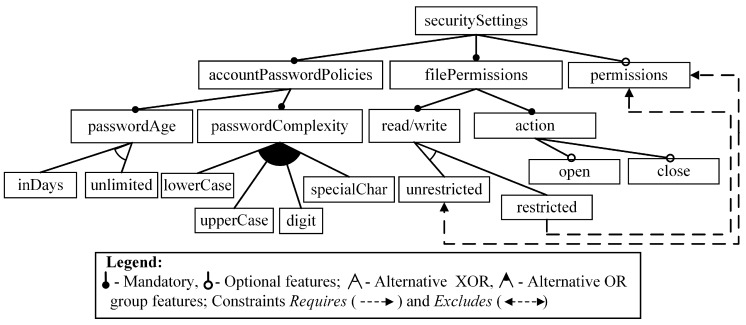
Motivating example to explain feature-based modeling concepts.

The methodology we use is based on: (1) representing both the problem domain (PD) and the solution domain (SD) by feature models and (2) mapping of the PD model onto the SD model using adequate transformation processes and rules. The PD model represents both non-functional (here identified as QoS) and functional requirements for the BAN software to be designed. The SD model specifies the heterogeneous meta-programming concepts and their relationships being represented at the highest level of abstraction. Firstly, we introduce a motivating example (Figure 2) to explain the basic concepts related to feature models [40]. Note that the example is not a part of our PD. In addition, this example serves for understanding the model characteristics to be obtained using the adequate tools (*i.e.*, FAMILIAR [41] and SPLOT [42]).

The feature-based notion is independent on any domain, including SD. However, when the concepts of the notion are defined, it is assumed that those concepts are projected on some abstract problem domain (*Definitions 1–6*). Note that the remaining *Definitions* of this section are SD specific.

*Definition 1.*  A feature is the distinguishing characteristic of a domain (system, component, process, requirement, *etc.*), which is important to consider by the stakeholder in the given context of use (for other definitions, see [36]). The feature is treated:

(*i*) as *mandatory features* that is always selected, if its parent feature is selected;
(*ii*) as *optional feature*s that can be selected or not;
(*iii*) as *alternative features* that are grouped and the selection from the group is governed by logical relations OR and XOR (see also Legend in Figure 2).

*Definition 2.*  A feature model (further FM, also *feature diagram*) is a compound of the following entities: root of the tree; set of edges of the type (i), (ii) and (iii) and constraints of the type *Requires* and *Excludes (Definition* 2 adapted from [41]).

*Definition 3.*  *A variation point* is the parent feature whose children are alternative or optional group features (e.g., the feature *passwordComplexity* in Figure 2). *A variant* (also atomic feature, see e.g., the feature *digit* in Figure 2) is the feature whose: (1) parent is a variation point and (2) which has no children.

*Definition 4.*  FM is said to be:

(i) *specialized* if it is derived from its ancestor FM through removing some features (if a parent feature is removed, all its children features are removed too);
(ii) *abstract* if some features have no atomic features with concrete values or, in the other context, some features may be decomposed into parts;
(iii) *concrete* if atomic features have the concrete values.

*Definition 5.*  FM *configuration* is the model that contains all *mandatory* nodes of the given FM, may contain optional nodes, and includes variation points, but the only one variant for each variation point is selected. A *valid configuration is* the configuration that takes into account the constraints (if any) among variation points and variants (e.g., there are 240 valid configurations in our example).

*Definition 6.*  *A variability model is* the sub-model containing variation points and variants of the FM. *The variability in space* is the number of the valid FM configurations*.*

**Figure 3:**
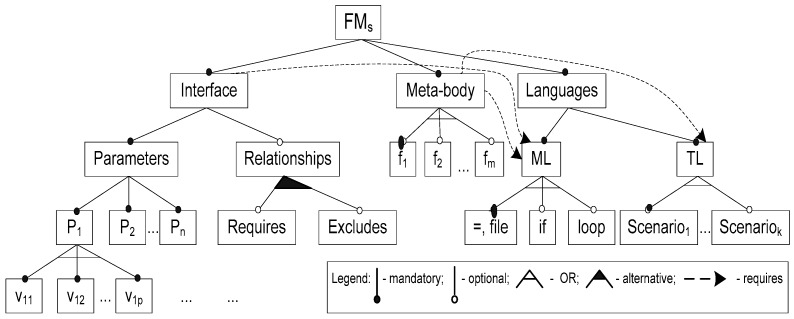
The solution domain feature model with abstract variants (shortly FMs).

The next definitions (adapted from [36]) define the basic terms related to the SD (*i.e.*, heterogeneous meta-programming). Furthermore, for better understanding of those terms, we introduce also an abstract FM to specify the SD (Figure 3).

*Definition 7.*  *Heterogeneous meta-programming* is the paradigm to express generic specifications using at least two languages at once: Meta-language (ML) and target (domain) language (TL). The first is a subset of the functions, called meta-functions, taken from a general purpose programming language (GPPL). It serves for expressing generalization through the external parameterization. The second is any domain-specific language (DSL) or GPPL and serves for expressing the base domain functionality through a domain program.

*Definition 8.*  *A meta-program* is the generic specification having the interface and meta-body. *An interface* is the set of prescribed parameters and their values. *A meta-body* is the implementation of the prescribed problem domain variability through the parameters and parameter-(meta-) function-target program relationships.

Note that the argument of a meta-function may be the parameter, the other function, the target program construct, or the combination thereof. In Figure 3, we summarize the meta-programming concepts using the feature-based notion. Here, the feature ‘Relationships’ means the possible parameter dependencies or interaction among their values (they are denoted as v_ij_ in Figure 3). It is possible to express the parameter relationship through constraints *requires* and *excludes* (see also *Definition 2*).

Now it is possible to describe the methodology in more detail.

### 5.2. Description of the Methodology

**Figure 4:**
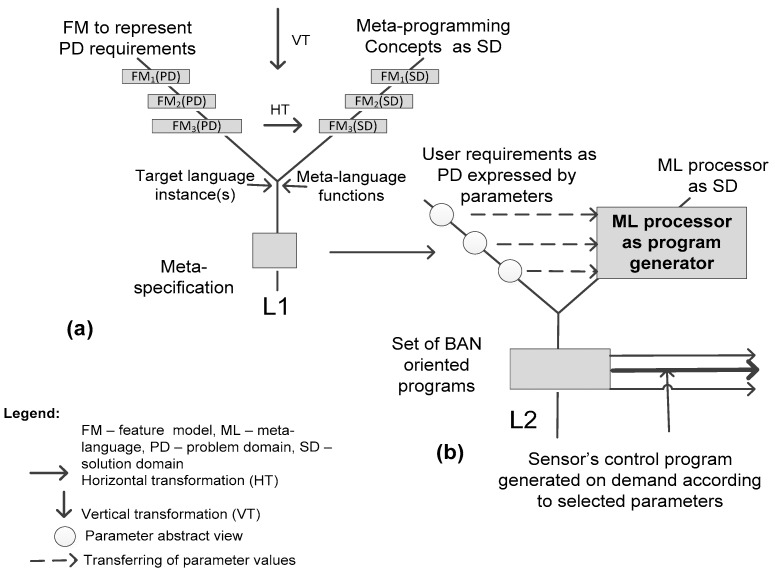
Principle of model-driven methodology: (**a**) transformation-based framework; (**b**) automatic design of BAN-oriented software.

We use the Y-chart to graphically illustrate the essence of the model-driven transformation, which in Figure 4a is presented abstractly as the mapping of the problem domain (PD) requirements onto the capabilities of the solution domain (SD). The requirements of the system are to be expressed formally, or semi-formally in order we could be able to check their consistency and correctness. As it was stated, we use feature models (FMs) to specify the PD requirements. The SD model also should be represented in the same notion to make the transformation possible.

We distinguish among vertical and horizontal transformations. By *vertical transformation* (*VT*) we mean the lowering the level of abstraction, *i.e.*, introducing more details in the model in order for it to be possible to first discover the transformation rules and then to apply them through the *horizontal transformation (HT)*.

First, we consider the VT. That is the *model-to-model* transformation that preserves the same abstraction level. For example, on the Y-chart left branch that represents the PD model transformations, notions FM_1_(PD), FM_2_(PD) and FM_3_(PD) mean the following VT:

FM_1_(PD)→FM_2_(PD)→FM_3_(PD),

where FM_1_(PD) is the initial aggregated abstract FM; FM_2_(PD) is the intermediate FM; FM_3_(PD) is the specialized concrete FM (see *Definition 4* in Section 5.1). Note that the VT is realized using the adequate feature modeling and verification tools (FAMILIAR and SPLOT). As the selected SD model is applied to the multiple problem domain tasks in the same way, the VT is not needed for the SD. However, to preserve the comprehensiveness in describing the methodology, we also present VT for the SD in the same way, but with the slightly different interpretation as follows:

FM_1_(SD)→FM_2_(SD)→FM_3_(SD),

where FM_1_(SD) is the abstract SD FM, containing the top part of the model given in Figure 3, *i.e.*, the following features: interface (parameters, relationships), meta-body, languages (ML, TL); FM_2_ (SD) is the intermediate abstract FM derived from FM_1_(SD) by adding the remaining upper features given in Figure 3; FM_3_(SD) is the concrete specialized SD FM derived from FM_2_ (SD) by substituting abstract feature variants with concrete values (e.g., ML→PHP, TL→C#, *etc.*). Now we consider the HT. The latter is defined as the two-level transformation process as follows:

(L1):  FM_3_(PD)→FM_3_(SD)→FM*_3_(SD)→*Meta-specification* (for the family of possible BAN applications);
(L2):  *Meta-specification*→BAN sensors control program or programs for concrete application.

Note that FM*_3_(SD) is derived from FM_3_(SD) by substituting abstract parameter values with the concrete ones taken from FM_3_(PD). Also, at the level L1, a target language should be identified to describe the PD (*i.e.*, the sensor’s functionality for sending and receiving data). The language is introduced in the process by selecting or creating a program instance (or instances, here treated as a part of the SD, see Figure 3) which are to be generalized using a meta-language and the PD variability model. The latter is extracted from the requirement feature model (*i.e.*, FM_3_(PD)). By applying the prescribed transformation rules, it is possible to create the meta-specification (the result of the transformation) either manually or using some transformation tool (e.g., given in [37]) as it is outlined in Figure 4a (see the level L1).

The meta-specification (aka meta-program) is the parameterized description. The parameters and fragments of a target language are arguments of the meta-language functions to perform various manipulations (it is treated as *horizontal transformation*) using the adequate transformation rules. For example, at the level L1, some items abstractly represented on the left branch as FM_3_(PD) should be thought of as *variation points* (along with variants) of PD FM. Some items of FM_3_(SD) on the right branch can be thought of as *parameters* of a meta-program to be created. Therefore, applying the predefined transformation rules, it is possible to create the meta-specification. Of course, there are more complex items (e.g., target language instance, meta-language functions on the right branch) and more complex transformation rules should be at hand in designing meta-specifications (those rules are not considered here).

The level L2 is for the application user. Again, we use the same visualization scheme. On the left branch, there is the meta-program interface (*i.e.*, parameters and their values are presented abstractly by circles). The application designer selects parameter values according to the specific requirements of the application. The meta-program implements the requirement variability space for the Smart Sensor Controller (SSC) that can be applied to a set of possible BAN applications. In our case study, the meta-program specification has been coded in two languages: PHP as a meta-language and C# as a target language. It was accepted that the BAN application should be realized just in the C# language. The PHP processor is the tool to automatically generate the BAN application programs (*i.e.*, SSC) on demand. Therefore, at the level L2, the tool serves as SD.

Now we are able to formulate the contribution of our approach in more details. Though at the model-to-model (M2M) transformation level our approach is similar to the known model-driven ones [8], it is novel in terms of the *model-to-program* (M2P) transformations. Typically, in known approaches by the program within M2P transformation, it is meant either the executable program, or its template (such as Visual Studio 2013 code). In our approach, the executable specification is the meta-program that enables us to automatically generate domain program instances on demand through the user defined parameter values. Therefore, our approach supports, to a larger extent, the reuse and adaptation capabilities.

Now we present the remaining part of the background, *i.e.*, transformation rules. We categorize them into two categories: (i) Model-to-Model (M2M) and (ii) Model-to-Program (M2P).

*Rule 1.*  Abstract FMs are obtained through domain analysis (DA) and modeling using the relevant DA approaches (such as FODA [35]) along with adequate tools (such as FAMILIAR [41] and SPLOT [42]).

*Rule 2.*  If an abstract model consists of separate models, then a model aggregation follows. The latter composes two or more input models without common features into the output model using the tool (such as FAMILIAR [41]).

*Rule 3.*  An abstract feature model is transformed into a concrete one by extending some lower-level features into features with concrete values so as to satisfy the design aims and requirements.

*Rule 4.*  A specialized model is derived (e.g., using the mentioned tools) from the concrete model under given specific requirements (such as narrowing the design space, *etc.*).

*Rule 5.*  The specialized concrete PD FM is transformed into the concrete SD FM using the abstract SD FM through mapping of corresponding PD items (e.g., variation points and variant) onto SD items (*i.e.*, meta-program interface and body) as it is defined by *Rules 6* and *7*.

Note that these rules are about M2M transformations that preserve roughly the same abstraction level. The rules are valid for both the PD and SD (for the latter, however, it is applied only once, because typically SD is common for different applications). Furthermore, these rules support both vertical and horizontal transformations (see Figure 4).

*Rule 6.*  The variation points of PD FM *correspond to parameters* within the meta-program or its model, and *variants* of a variation point *correspond to the parameter value.*

We explain this rule in detail: In case of realizing our motivating example, an abstract parameter name (say P1, see Figure 3) is substituted by the concrete variation point (say, *passwordAge*, see Figure 2). Also, an abstract parameter value (say, v_11_) is substituted by the variant (say, *inDays*).

*Rule 7.*  The parameter and their values are to be specified in the interface of the meta-program. The dependences among parameter values (if any) are to be *specified* in the interface too and expressed through the constraints (*requires*, *excludes)* to be implemented by the alternative meta-function (such as *if*-function of the meta-language).

For instance, the realization of the rule in a pseudo-code for ‘*requires’* in our motivating example might look as follows:

**if***read/write***is equal to***restricted***then***permissions***is** true;

This example also illustrates the essence of HT (see Figure 4), though abstractly and in a very simplified mode. We present a real implementation of HT in our case study.

*Rule 8.*  The SD model (*i.e.*, meta-program model) is transformed into the executable meta-program specification (MPS) by performing the following actions: (i) selecting the concrete meta-language (ML) constructs; (ii) choosing the relevant target language (TL) scenario or scenarios (that depends on the task complexity); (iii) generalizing them using the ML constructs and PD variability model through coding and testing the specification.

*Rule 9.*  The executable MPS is transformed into the application programs (such as the BAN controller in our case) via the following actions: (i) selecting a pre-programmed parameter values taken from the interface; (ii) processing (interpreting) MPS by the ML processor and generating the concrete target program; (iii) adapting it to the different use cases (if any) by the re-generation process.

Note that Rules 6–9 are about M2P transformations. The latter changes the abstraction level, lowering it. The rules are the background to understand the processes we abstractly outlined in Figure 4. The formal definition of our approach, however, was not the intention of this paper. A more deep theoretical reasoning can be found in the numerous other publications (e.g., also in those we have cited with regard to FM transformations [35,40,41] and meta-program transformations [36,37]).

## 6. Case Study and Results

The case study includes two sections. In Section 6.1, we present some hardware-software implementation results with regard to energy-security issues in the IoT obtained through physical modeling and provided measurements. In Section 6.2, we describe the development of the generic specification (*i.e.*, meta-program) to cover a variety of the sensor controller programs for the multiple BAN applications.

### 6.1. Results on Hardware-Software Implementation

**Figure 5:**
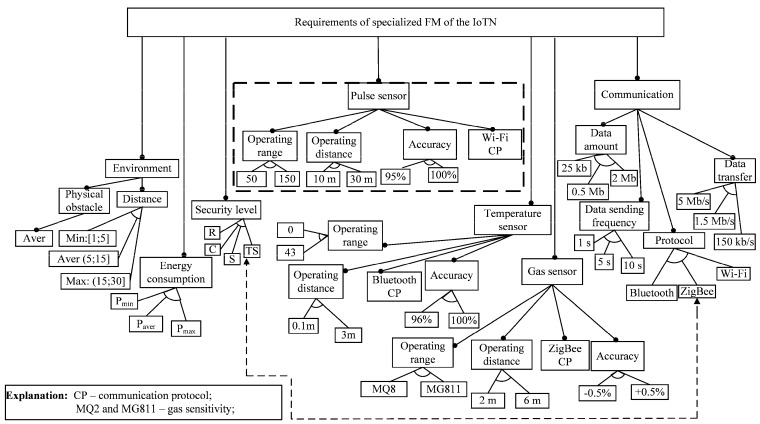
Specialized concrete feature model to specify generic requirements for a set of BAN applications.

In Figure 5, we present the requirements specification for the IoT node (IoTN) using feature-based notation (see also Section 5.1). The model has been derived from the statement of initial requirements given by the domain expert. The background of doing so is Rules 1–4 (see Section 5.2). The model is a specialized concrete feature model (see *Definition 4* in Section 5.1), which is restricted by specifying the requirements of three dedicated sensors for the pulse, temperature and gas measurements (so is done for narrowing the possible solution space). The constraint relationships are given separately in Table 1 due to the better readability of the model. The model characteristics obtained using the FAMILIAR and SPLOT tools [41,42] are given in Table 2.

**Figure 6:**
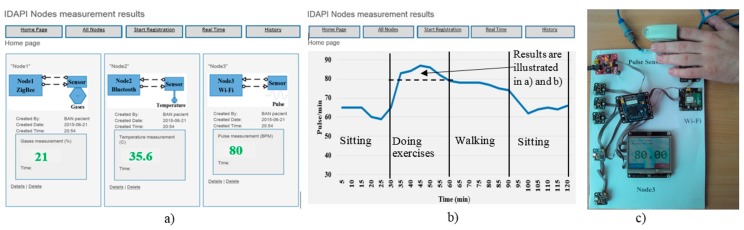
The views: (**a**) real time measurements (both on the patient’s side) and (**b**) the pulse signal history for monitoring over the Internet in the remote station; (**c**) the implemented hardware of the pulse sensor.

In Figure 6, we outline the view of the hardware implementation along with the sensor measured data (see also the dash line rectangle in Figure 5) obtained in real time mode. Three control programs (in C# language) to read and send data from the sensor were developed and tested taking into account the concrete characteristics of the sensors. The provided experiments proved the correct functioning of the proposed network model. The control programs were developed manually and required a great deal of designer efforts. Therefore, this fact was the stimulus to automate the process as it is presented in the second part of this case study.

### 6.2. Results of BAM Meta-Program Implementation

**Figure 7:**
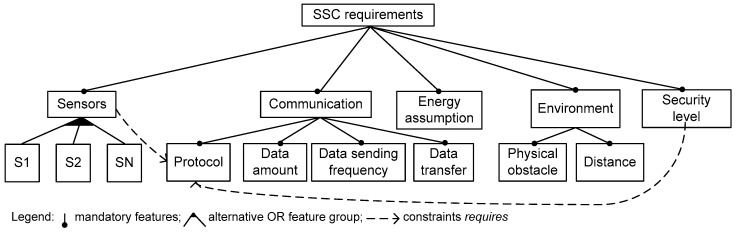
Abstract feature model to specify requirements of BAN-related applications.

**Figure 8:**
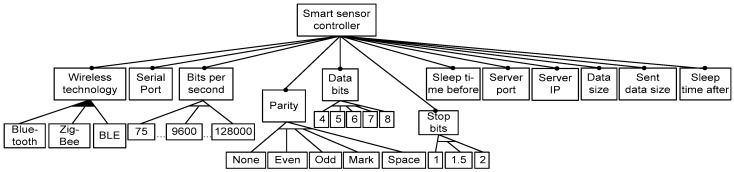
SSC implementation-level concrete feature model.

In this part of the case study, we have used the tested control programs as instances to develop the generic specification (meta-program). They were obtained as a result of experiments described in Section 6.1. Firstly, in Figure 7, we present requirements as an abstract FM to design the SSC for BAN applications. In Figure 8, we present the implementation level of the SSC feature model as the initial specification to develop the generic specification. To derive the models, we have used Rules 2–4. The characteristics of the model are given in Table 3. Note that, one configuration in the feature model corresponds to the sensor’s control program, when the FM is implemented correctly. Therefore, as the configuration count of the FM is at least equal to 9450 (see Table 3), we are able to derive the same number of sensor’s control programs from the generic specification. They differ by parameter values (they are seen as feature variants of the grouped features in Figure 8).

**Figure 9:**
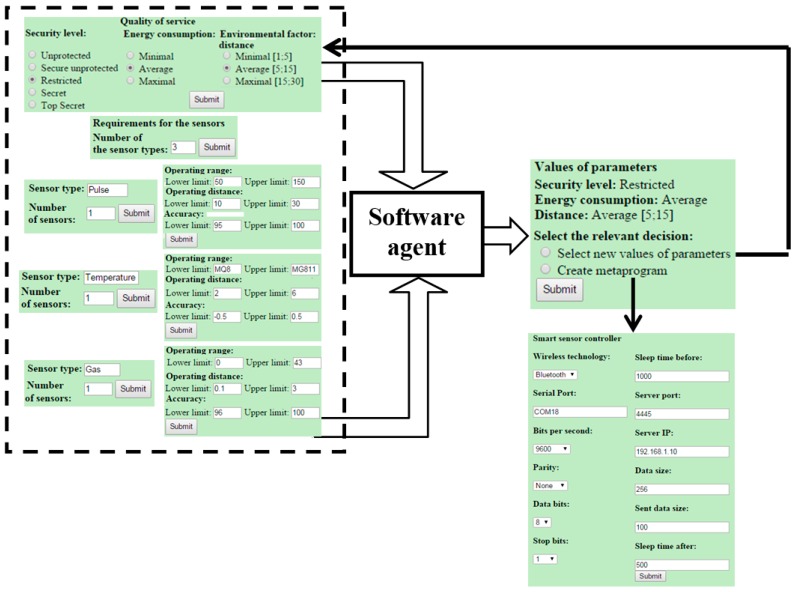
Structure of the two-level meta-program interface.

The concrete feature models (Figure 5 and Figure 8, see also Section 5.1) are the solution of task 1 and also the high-level specification to deal with task 2 (see Section 3). Using this specification, we have developed the meta-program using Rules 5–9 (see Section 5.2). The interface is shown in Figure 9. The top-level of the interface (on left) serves for the system designer to select the initial QoS and sensors’ parameter values. The body of the top-level meta-program is fully hidden from the designers. The essential part of the body is the call to the software agent. The latter accepts the submitted data and decides, whether it is possible to satisfy the requirements, or not. If not, the software agent indicates on miss-matched parameter values and returns this information to the system designer for making the correction. If the requirements are valid, the identified parameter values are transferred to the lower-level interface (see boxes on the right). Having those values, the PHP processor generates the adequate variant of the control program for the SSC automatically.

Note that we use a set of PHP functions as a meta-language to specify the generalization through parameterization. Therefore, the PHP processor is the control program generator (aka meta-program). We use C# as a target language (assuming that the BAN software system should be realized in that language) to specify the base SSC functionality (see also Section 5.2). Note also that the development of the software agent is not the subject of this paper. We summarize the characteristics of the developed meta-program in Table 4.

**Figure A1:**
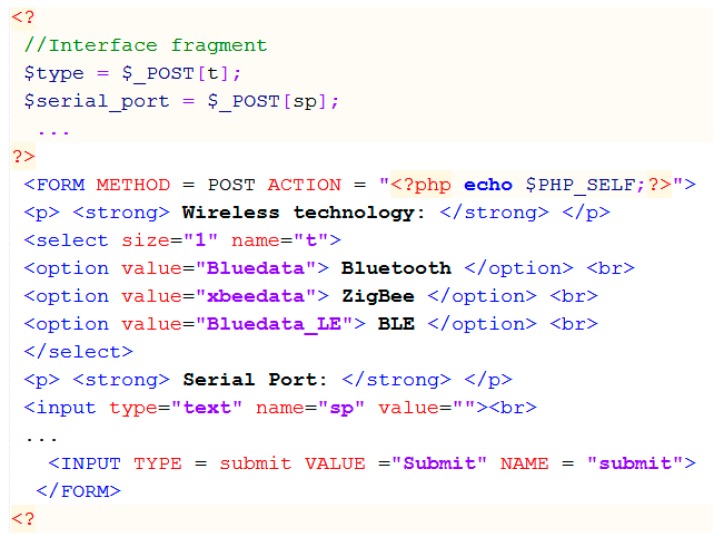

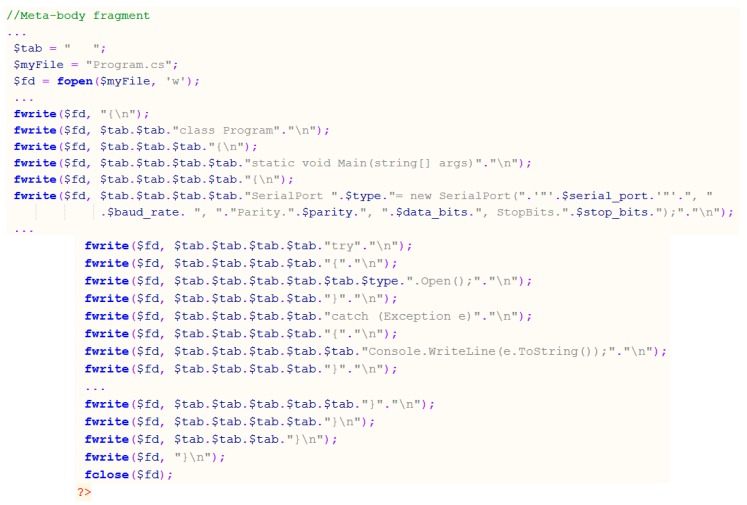
Meta-program fragments in PHP, using HTML for representation of parameters and their values.

**Figure A2:**
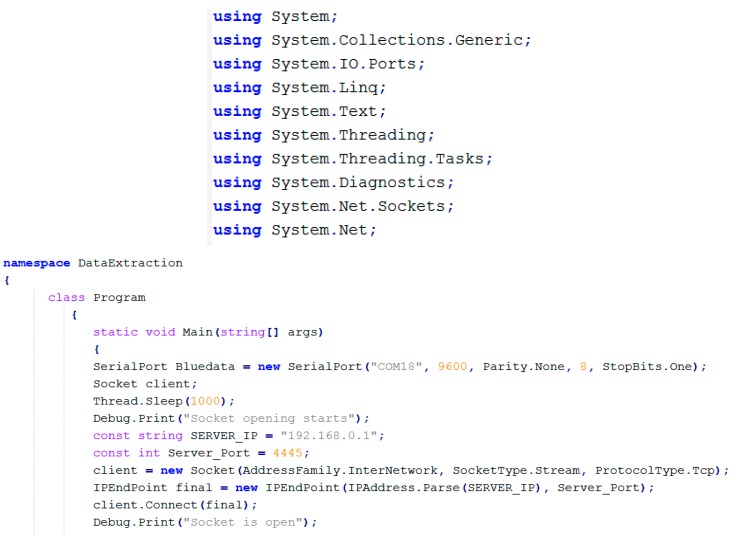

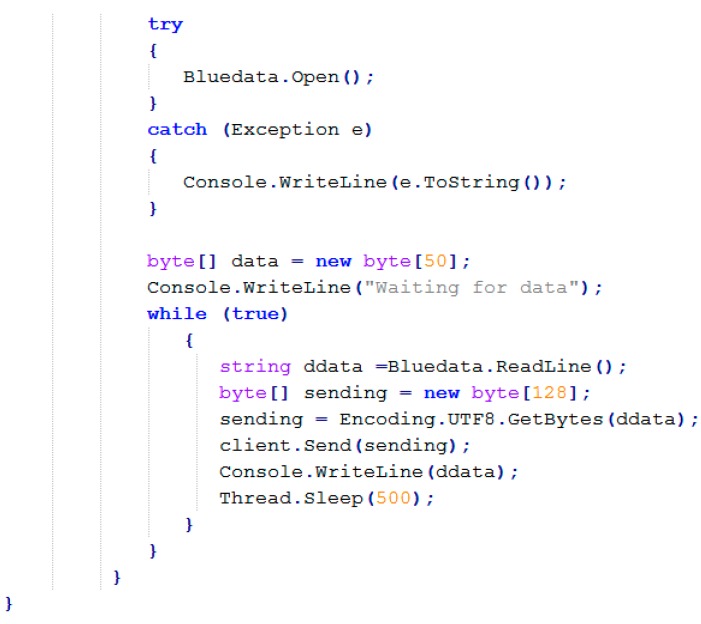
Generated instance in C# for smart sensor controller of BAN.

We provide more details on the developed specifications in Appendix A. In Figure A1, we present a fragment of the meta-program written in PHP. Note that for representing parameters and their values (see the bottom box in Figure 9 on right) also HTML is to be used. For other details, see comments within the specification (Figure A1). In Figure A2, we present the generated program (instance) in C# for data transferring. The parameter values used for generating the instance are also seen in Figure 9.

## 7. Discussion and Evaluation

IoT-oriented healthcare applications, among others, have been widely discussed in recent years. Body Area Networks (BANs) are seen as an important segment of those applications. In this paper, we have introduced a BAN-oriented IoT *model (prototype*) as the two-layered architecture containing the standard Internet and application modules at the top layer and the IoT nodes in the lower layer. The node of the IoT is a set of the smart sensors mounted on the human’s body along with the smart controller for data acceptance, decision making and control of data transfer in both directions: from and to smart sensors and from and to the application level. BAN is a net of the IoT nodes. The whole functionality of the applications depends highly on the quality and timelessness of the initial data collected from the human’s body by the BAN and then transferred to the top level for evaluation and management. The quality of service (QoS) is predefined, to the largest extent, by the strict requirements for energy resources, security and environmental factors. As those issues are very dependent, it is extremely important to understand and represent those relationships at the early stage of the BAN development, *i.e.*, at the stage of specifying requirements.

Feature-based modeling in analysis of the requirements is regarded as the relevant approach in developing systems for many other applications. However, in the case of BAN development, this approach to our best knowledge, either has not been exploited at all yet or there are only incipient attempts in this direction. Therefore, in this paper we have introduced the concept of feature-based modeling and applied them in designing software for BAN applications to achieve two objectives: (1) showing the relevance of feature-based notion in specifying multiple aspects of relationships for analysis and the whole understanding of the requirements and system functionality at the early stage; (2) using specialized feature models for automated software design.

We have discussed the methodology that has three significant parts: (1) requirements specified at a higher abstraction level using feature models for the IoT and BAN applications; (2) generative technology as a solution domain also presented by feature models; (3) seamless integration of both models through model transformations to achieve the goals of automation. Though the parts (2) and (3) are more related to the interests of IoT software developers, part (1) may be useful to the much larger community, such as the IoT domain policy makers, the IoT researchers and the practitioners. This is so because the high-level feature modeling introduces and supports the systematic vision of complex items relationships and constraints in the human readable form.

We have evaluated the proposed methodology by presenting a case study. The latter includes two parts. In the first part, we have presented the hardware-software implementation of the simplified concrete BAN structure (*i.e.*, prototype) and some experiments we have carried out. Taking from the experimental system the sensors’ control programs and applying the proposed model-driven methodology in the second part, we have developed the generic meta-specifications to specify the functionality of the smart sensor controller for a variety of possible BAN applications. The meta-specification has been developed using PHP as a meta-language. It is possible to automatically generate the sensor controller’s control programs on demand for a concrete BAN application, using this specification and the PHP processor. The number of such programs depends on the number of parameters and their values that were incorporated into the specification. This number may be huge, e.g., in our case study, it may exceed 9450. Therefore, the designer of a BAN application has a huge space for selecting the most relevant solution automatically. In doing that, *i.e.*, instead of writing the program manually, he or she needs to do only one thing—indicate the concrete parameter values.

## 8. Conclusions

A great deal of IoT applications are concerned with healthcare. The typical case is the BAN-oriented application that is based on using WSNs. The development of those applications is hard due to the following reasons: (a) the need to satisfy not only the functional requirements for data collection and management, but also to ensure the strict requirements for non-functional requirements (energy, security, privacy, *etc.*); (b) applications cover both hardware and software parts; (c) typically the software part is unique and requires essential human efforts and therefore is complex. The relevant solution as the response to the emerging challenges is the use of prototyping combined with modern model-driven methodologies in designing the systems. The feature modeling approach used in this research allows one to extract and represent the requirements at the early stage of the development for analysis, evaluation and introduction of changes before implementation. Furthermore, the specialized feature models to specify BAN application requirements, combined with the generative technology, such as meta-programming, enables one to automatically generate software for a family of related BAN applications. Having the generic specification (such as the one demonstrated in our case study to describe the smart sensor controller), it is possible to make the decision and receive the final solution (*i.e.*, executable control program) (i) *as fast as possible* and (ii) to obtain the *acceptable degree* of the functionality (in terms of quality of service of the system to be designed, meaning also a Pareto-optimal solution) through the *interactive adjustment of parameter values and re-generation process* in adapting the requirements for the concrete application.

## Figures and Tables

**Table 1:** Constraint relationships within the feature model (Figure 5).

No.	Features	Constraints	Features
1	Pulse Wi-Fi	*Requires*	Wi-Fi
2	Gas ZigBee	*Requires*	ZigBee
3	Temperature Bluetooth	*Requires*	Bluetooth
4	25 Kb	*Requires*	150 kb/s
5	2 Mb	*Requires*	5 Mb/s
6	0.5 Mb	*Requires*	1.5 Mb/s
7	Wi-Fi	*Requires*	5 Mb/s
8	Bluetooth	*Requires*	1.5 Mb/s
9	ZigBee	*Requires*	150 kb/s
10	Bluetooth	*Requires*	C
11	ZigBee	*Excludes*	TS
12	TS	*Requires*	Wi-Fi
13	Pulse sensor	*Requires*	Max: (15; 30]
14	Gas sensor	*Requires*	Aver: (5; 15]
15	Temperature sensor	*Requires*	Min: [1; 5]
16	Pulse Wi-Fi	*Requires*	Pmax
17	Temperature Bluetooth	*Requires*	Paver
18	Gas ZigBee	*Requires*	Pmin
19	Pulse sensor	*Requires*	1 s
20	Gas sensor	*Requires*	5 s
21	Temperature sensor	*Requires*	10 s
22	Pulse Wi-Fi	*Requires*	2 Mb
23	Temperature Bluetooth	*Requires*	25 kb
24	Gas ZigBee	*Requires*	0.5 Mb

**Table 2:** Basic metrics of the SC feature model taken from the tool SPLOT.

Characteristics	Specialized FM (Figure 5) of the Prototype (Figure 3)	FM of the Motivating Example (Figure 2)
#Features	69	18
-Optional	0	3
-Mandatory	18	6
-Grouped	40	8
-Groups	16	3
Tree Depth	5	4
#Extra constraints	24	2
**Debugging Analyses**		
#Dead Features	0	0
Count Configurations	8	240

**Table 3:** Basic metrics of the SSC feature model taken from the tool SPLOT.

**Characteristics**	**SSC FM**
#Features	47
-Optional	0
-Mandatory	12
-Grouped	34
-Groups	5
Tree Depth	3
#Extra constraints	0
**Debugging Analyses**	
#Dead Features	0
Count Configurations	Since 9450

**Table 4:** Basic metrics of meta-program and its instances for BAN-oriented applications.

No.	Criteria	Value	Properties
1	Meta-language	PHP	May be used another language
2	Target language	C#	May be used another language
3	Meta-program size	11.7 kB	
4	The average size of the generated program	1.56 kB	
5	# of instances that can be generated	Since 9450	Depends on the number of parameters and their values
6	# of tested variants of generated programs	15	Covers all critical points of meta-program
7	# of parameters	24	All parameters are independent
8	# of different meta-functions	5	Functions that perform simple operations (fopen, fwrite, fclose), conditional function (if)
9	Total # of meta-functions	69	Allows to evaluate the complexity of meta-program
10	Total # of parameters	329	Defines cognitive understandability of the meta-program
11	Relative Kolmogorov’s Complexity (RKC). A high value of RKC means that there are fewer capabilities for compression, *i.e.*, there are less repeating parts within the meta-program, and therefore this meta-program is regarded as being less complex.	0.22	A ratio of the size of the compressed meta-program using a compression algorithm BWT (Burrows-Wheeler transform, see GnuWin32) to the size of the initial meta-program [33].
